# Guanidine Acetic Acid Alters Tissue Bound Amino Acid Profiles and Oxidative Status in Finishing Pigs

**DOI:** 10.3390/ani13101626

**Published:** 2023-05-12

**Authors:** Yiyan Cui, Miao Yu, Zhenming Li, Min Song, Zhimei Tian, Dun Deng, Xianyong Ma

**Affiliations:** 1 State Key Laboratory of Livestock and Poultry Breeding, Institute of Animal Science, Guangdong Academy of Agricultural Sciences, Guangzhou 510640, China; 2 The Key Laboratory of Animal Nutrition and Feed Science in South China, Ministry of Agriculture, Guangzhou 510640, China; 3 Guangdong Provincial Key Laboratory of Animal Breeding and Nutrition, Guangzhou 510640, China; 4 Guangdong Engineering Technology Research Center of Animal Meat Quality and Safety Control and Evaluation, Guangzhou 510640, China; 5 Maoming Branch, Guangdong Laboratory for Lingnan Modern Agriculture, Maoming 525000, China

**Keywords:** guanidine acetic acid, pig, amino acid, *longissimus thoracis*, myocardium

## Abstract

**Simple Summary:**

This study aims to investigate the effects of GAA on carcass traits, plasma biochemical parameters, tissue antioxidant capacity, and tissue amino acid contents in finishing pigs. The results of this study indicate that GAA enhanced the plasma biochemical parameters, oxidative status, and amino acid profiles of the heart and *longissimus thoracis* muscle in finishing pigs.

**Abstract:**

This study aims to investigate the effects of guanidine acetic acid (GAA) on carcass traits, plasma biochemical parameters, tissue antioxidant capacity, and tissue-bound amino acid contents in finishing pigs. Seventy-two 140-day-old (body weight 86.59 ± 1.16 kg) crossbred pigs (Duroc × Landrace × Large White) were randomly assigned into four treatments with six replicate pens and three pigs per pen, which were fed the basal diets supplemented with 0, 0.05%, 0.10%, or 0.15% GAA, respectively. The plasma glucose concentration decreased, and creatine kinase activity and levels of GAA and creatine increased with the dietary GAA concentration. GAA linearly improved creatine content in the *longissimus thoracis* muscle (LM) and heart. The activities of superoxide dismutase, total antioxidant capacity, and glutathione peroxidase increased linearly in tissue or/and plasma, while the contents of malondialdehyde and protein carbonyl decreased linearly. GAA improved the contents of multiple-bound amino acids (such as proline or isoleucine) in the myocardium and LM. In conclusion, GAA enhanced the plasma biochemical parameters, oxidative status, and bound amino acid profiles of the heart and LM in finishing pigs.

## 1. Introduction

Guanidine acetic acid (GAA) is an endogenous substance of creatine biosynthesis that is synthesized from glycine and arginine [1]. Supplementation of GAA could prevent the biosynthesis of GAA from arginine and glycine to increase the creatine content in the body. This allows more arginine, glycine, and creatine to be used for the biosynthesis of amino acids and proteins to encourage animal growth, which enhances animal production. Previous research has shown that dietary GAA improves the growth performance of male broilers and pigs [2,3,4], as well as the meat quality of pig (grower, finisher), broiler, and growing grass carp [5,6,7,8]. We have shown that supplementation with GAA alters the free amino acid profiles of plasma and tissues in finishing pigs [9]. However, no studies to date have characterized GAA’s tissue-bound amino acid profile and whether GAA also alters the body’s tissue protein-bound amino acid composition in finishing pigs. Animal body proteins are continuously synthesized and degraded in all tissues [10]. Dietary strategies can increase protein synthesis or improve protein breakdown [11]. Visceral tissue accounts for approximately 11% of monogastric animal protein mass and accounts for 35% of body protein biosynthesis [12]. The liver, kidney, pancreas, heart, and spleen affect the nutrient supply and systemic metabolism. Skeletal muscle is the largest protein pool. Tissue proteins are composed of various kinds of proteins with different amino acid compositions. If amino acid profiles of muscle and other tissue proteins are influenced by GAA supplementation, carcass traits may also be altered. In this study, we measured the effects of dietary GAA on carcass traits, plasma parameters, antioxidant capacity in plasma and tissue, and tissue-bound amino acid profiles in finishing pigs to aid the application of GAA supplementation in animal husbandry.

## 2. Materials and Methods

Animal procedures and experiments were approved by the Animal Care and Use Committee of the Guangdong Academy of Agricultural Sciences (authorization number GAASIAS-2019-02-21).

### 2.1. Experimental Design, Animals and Diets

The study was conducted in the pig farm of the Institute of Animal Science, Guangdong Academy of Agricultural Sciences (Guangzhou, China). Seventy-two 140-day-old (body weight 86.59 ± 1.16 kg) crossbred pigs (Duroc × Landrace × Large White) were randomly assigned into four treatments with 6 replicate pens, and 3 pigs per pen, which were fed the basal diets supplemented with 0, 0.05%, 0.10%, or 0.15% GAA, respectively. GAA (99% purity) was provided by Guangdong Newland Feed Science Technology Co., Ltd. (Guangzhou, China). All diets were formulated to meet or exceed the requirements suggested by the NRC 2012, and the ingredients and nutrient composition of the basal diet were the same as that of our previous study [9]. All pigs were given free access to feed and water during the 42 days.

### 2.2. Slaughter Procedure and Sample Collection

The slaughtering procedure and sample collection followed the procedures described in the study of Cui et al. [9]. Briefly, the live weight of pigs was determined before slaughtering. After fasting for approximately 12 h, blood was collected, and the pigs (6 pigs from a group) were slaughtered according to commercial procedures in the slaughterhouse of the Institute of Animal Science, Guangdong Academy of Agricultural Sciences (Guangzhou, China) in 43rd day of the test. Plasma, heart, liver, spleen, pancreas, kidneys, and *longissimus thoracis* muscle (LM) were collected, frozen in liquid nitrogen, and then stored at −80 °C.

### 2.3. Carcass Traits

The carcass weight and abdominal fat weight were measured. The average back fat thickness was determined at 1st rib, lumbar, and last rib of the left side. The loin muscle area was determined with a digital planimeter (KP-90N, Koizumi, Sapporo, Japan).

### 2.4. Biochemical Plasma Parameters

The contents of plasma glucose (000020100), albumin (000020150), total protein (000020140), creatinine (000020170), urea (000020070), uric acid (000020110), and the activities of alanine aminotransferase (000020000), aspartate aminotransferase (000020010), creatine kinase (000020040) were measured using a biochemical analyzer (Vital Selectra ProXL, Beijing, China) according to the manufacturer’s instructions (BIOSINO, Beijing, China).

### 2.5. GAA, Creatine, and Chemical Composition Analysis

According to Wang et al. [13], the GAA in plasma and tissues was separated by a high-performance liquid chromatography (UltiMate 3000, Thermo Scientific, Waltham, MA, USA) eluted with 0.02 mol/L dipotassium hydrogen phosphate and acetonitrile buffer (40:60), at a flow rate of 1 mL/min for 10 min. Creatine content was determined with a kit (BC4925, Beijing Solarbio Science & Technology Co., Ltd., Beijing, China) at 530 nm (Spectra Max M5, Molecular Devices, San Jose, CA, USA). According to Cui et al. [14], the contents of moisture, crude protein, lipid, and ash were analyzed by the freeze-drying method, Kjeldahlmethod, Soxhlet extraction, and burning method, respectively.

### 2.6. Antioxidant Index Analysis

Antioxidant index in tissue was measured after the homogenization: approximately 0.1-g tissue samples were homogenized in saline (1:9, wt/vol) and then centrifuged at 3500× *g* for 15 min at 4 °C. The tissue supernatant was obtained. Activities of superoxide dismutase (SOD; A001-1-2), total antioxidant capacity (T-AOC; A015-1-2), and glutathione peroxidase (GSH-Px; A005-1-2), and the content of malondialdehyde (MDA; A003-2-2), protein carbonyl (A087-1-2), and total protein (A045-2-2) in tissues supernatant and plasma were determined by various kits (Nanjing Jiancheng Bioengineering Institute, Nanjing, China) with a multi-functional enzyme labeling instrument (Spectra Max M5, Molecular Devices, San Jose, CA, USA).

### 2.7. Hydrolyzed Amino Acid Analysis

The freeze-dried samples were dissolved in 6 mol/L hydrochloric acid at 110 °C for 24 h. Next, a 1 mL diluted sample (diluted to 50 mL) was evaporated at 60 °C. The residue was reconstituted with 0.02 mol/L hydrochloric acid and filtered with a 0.45-μm membrane. The content of amino acids was analyzed by an automatic amino acid analyzer (L-8900, Hitachi Ltd., Tokyo, Japan). Total essential amino acids (EAA) and non-essential amino acids (NEAA) were calculated.

### 2.8. Statistical Analysis

Pigs were the experimental unit. The significance of differences among groups was determined using one-way ANOVA followed by Tukey’s multiple range test with SPSS 25.0 (SPSS Inc., Chicago, IL, USA). Linear and quadratic contrasts were estimated with increasing GAA. The results were expressed as means and standard error of the mean (SEM). *p* < 0.05 was considered significant, whereas 0.05 < *p* ≤ 0.10 was considered a trend.

## 3. Results

### 3.1. Carcass Traits

The loin muscle area had a linear increasing trend with the level of GAA supplementation (*p* = 0.084) (Table 1).

### 3.2. Biochemical Plasma Parameters

The concentration of glucose (linear and quadratic, *p* < 0.05) decreased, but the concentration of GAA and creatine and creatine kinase activity increased significantly (linear or quadratic, *p* < 0.05) as the level of GAA supplementation increased (Table 2). Increasing GAA supplementation had linear and quadratic effects on the content of albumin (*p* = 0.047).

### 3.3. GAA and Creatine Contents in Longissimus Thoracis Muscle and Heart

Dietary GAA did not alter GAA concentration in LM and heart (*p* > 0.05) but linearly increased creatine content in LM and heart (*p* < 0.05) (Table 3). Dietary GAA did not alter chemical composition in LM (*p* > 0.05) (Table S1).

### 3.4. Antioxidant Capacity

With the level of GAA supplementation, the activities of SOD (except pancreas; *p* < 0.05), T-AOC (plasma, spleen, heart, and LM; *p* < 0.05), and GSH-Px (spleen, heart, and LM; *p* < 0.05) increased linearly, while the contents of MDA (plasma and spleen; *p* < 0.05) and protein carbonyl (plasma and tissue; *p* < 0.05) decreased linearly (Table 4). However, variation in the level of GAA did not significantly alter pancreatic antioxidant enzyme activity (*p* > 0.05).

### 3.5. Amino Acid Profiles

No significant difference in amino acid content in liver, kidney, pancreas, or spleen (*p* > 0.05; Tables S2–S5) was observed.

As shown in Table 5, increasing GAA supplementation significantly increased the contents of proline, alanine, aspartic acid, and NEAA linearly (*p* < 0.05) in the heart and had quadratic effects on the concentrations of methionine, isoleucine, leucine, tyrosine, and EAA (*p* < 0.05), and tended to have linear or quadratic effects on the concentration of valine, lysine, phenylalanine, histidine, arginine, serine, glutamate, glycine, and total amino acids (0.05 < *p* ≤ 0.10).

As shown in Table 6, increasing GAA supplementation had significant effects on the contents of threonine, methionine, isoleucine, carnosine, and anserine (linear and quadratic, *p* < 0.05) in LM and tended to have quadratic effects on the contents of leucine, lysine, arginine, proline, serine, glycine, alanine, aspartic acid, tyrosine, EAA, and NEAA (0.05 < *p* ≤ 0.10).

## 4. Discussion

GAA supplementation has beneficial effects on carcass traits [5,15]. The addition of exogenous GAA increased loin muscle area, which is consistent with previous studies, showing that supplementation with 0.06% GAA increases lean muscle mass and loin muscle in pigs but has no significant effect on carcass weight and carcass yield [5]. GAA increases creatine biosynthesis, provides extra energy, and promotes the deposition of muscle protein [16]. However, the effect of GAA on carcass performance varies among studies; it might be related to treatment time (60 or 42 d), animal physiological state (stressed or normal), and animal genotype (Duroc × Landrace × Yorkshire or Duroc × Landrace × Large White).

Blood biochemical parameters reflect animal physiological conditions. Creatine kinase is a central regulatory enzyme of energy metabolism and is usually found in tissues that require high-energy (heart and skeletal muscle) [17]. In our study, the creatine kinase activity [7,18,19], the contents of GAA [4,20,21,22], and creatine [4,21,22,23,24] of the GAA group were significantly increased, which was consistent with previous findings. GAA is absorbed by the intestine and then distributed to other parts of the body, increasing creatine kinase activity, contents of GAA, adenosine triphosphate, and creatine [7,19], and reducing the concentration of glucose for energy [25], indicating that GAA improves the energy state of pigs. This also confirms the findings of Liu et al. [25]; GAA reduces energy expenditure by providing extra energy in vivo [25]. The elevated level of creatinine is considered to be an impairment of the kidney [26]. Dietary GAA increases the blood creatinine concentration in cattle [22,27], chickens [7], and fish [19]. However, in our study, GAA did not increase the plasma creatinine content, which is consistent with previous results [4], suggesting that GAA has no detrimental effect on the renal function of finishing pigs.

Dietary supplementation with GAA can improve the oxidative status of pigs [28], humans [29], cattle [27], sheep [30], chickens [31], quail [32], and Nile tilapia [20]. The activities of antioxidant enzymes in tissues (except pancreas) and plasma increased with the dietary GAA concentration, while the content of protein carbonyl in tissues and plasma decreased, which indicated that the oxidative status of finishing pigs improved with GAA concentration. The addition of amino acids and their derivatives may improve antioxidant enzyme activity by supporting the basal activity needed to maintain circulation [33]. The antioxidant enzyme activities of the pancreas did not increase. Interestingly, GAA linearly reduced carbonyl content in the pancreas. Organs vary in their responses to GAA, which might be related to the levels of tissue-free amino acids. In a previous study, GAA changed the free amino acid content in these viscera; GAA also increased the free amino acid levels in the spleen and decreased it in the pancreas [9]. Carnosine and anserine have antioxidants, muscle contractility, and metabolism-regulating effects [34]. GAA increased the levels of carnosine and anserine in LM, which might be related to the increased antioxidant capacity of LM. Whether and how GAA improves oxidative stress remains unknown. GAA enhanced creatine levels in plasma, LM, and heart, which was consistent with Ostojic [35], who reported that GAA increased the creatine level in muscle and the whole body. This demonstrates that the increases in the plasma creatine concentration are related to increases in the creatine concentration in tissues, and GAA supplementation promotes tissue growth. Studies have shown that GAA administration can increase the level of creatine in tissues and enhance the activity of antioxidant enzymes in the body [34,36]. GAA is indirectly useful as an antioxidant due to the free radical scavenging ability of its metabolites, creatine, and arginine [32]. In addition, pig viscera are widely consumed in China. The improvement of oxidative status is not only conducive to the health of animals but also conducive to prolonging the shelf life of these visceral foods.

Isoleucine and leucine could stimulate protein synthesis and inhibit proteolysis [37]. The main functions of methionine are protein synthesis, oxidative stress inhibition, trans-methylation, and trans-sulfuration [38]. Changes in isoleucine, leucine, and methionine in the heart and LM may be beneficial for modulating cardiac and LM signaling pathways such as mTOR signaling [39,40].

In skeletal muscle, the balance between amino acid catabolism and anabolism is complex due to the metabolic and anabolic flux of muscle fibers [41]. GAA tended to increase the content of most amino acids in LM because the protein synthesis and degradation are balanced under physiological conditions, and the size of the muscle fibers remains the same (the crude protein content in LM was similar under different concentrations of GAA, unpublished data). The most important goal in the production of finishing pigs is to produce muscle to provide meat for humans. Amino acid is the basic composition of protein, and its composition is considered an important parameter for estimating pork’s nutritive value. GAA increased the amino acid level of LM, especially threonine, methionine, and isoleucine. The total amino acids of LM in pigs fed with different concentrations of GAA remained relatively stable, but the content of EAA and NEAA increased. GAA enhanced pork’s nutritive value, which is good for human health. In addition, the enhanced antioxidant capacity of LM increases the shelf life of pork.

Our research and previous research found that GAA could enhance the muscle creatine content [42,43]; however, it has no effect on the muscle GAA concentration [4,24]. GAA supplementation cannot increase GAA content in muscle might stem from the fact that GAA is mainly transported to liver tissues for creatine synthesis [35] rather than tissues with accumulated creatine [44]. The creatine/creatine kinase system plays a key role in cellular energy buffering and transport, particularly in cells with high and fluctuating energy requirements, such as in skeletal and cardiac muscle, brain, and spermatozoa [45]. The muscle is the main organ of creatine storage in animals [46]. Increases in the creatine content improve the energy metabolism of cells in energy-demanding tissues (LM and heart). Creatine is converted to creatine phosphate to improve muscle performance, maintain muscle energy reserves, and possibly improve muscle quality. The increase in the creatine content explains some of the health benefits (improvement of oxidative status and amino acid composition) of GAA supplementation in finishing pigs.

GAA enhanced amino acid levels of the LM and heart. GAA promotes the deposition of proteins by mediating the effect of creatine. When the concentrations of amino acids are sufficient, supplementation of GAA leads to the deposition of different types of proteins; thus, muscle is strongly affected by GAA, whereas the liver, kidney, pancreas, and spleen are weakly affected by GAA supplementation. GAA alters amino acid metabolism in pigs [9]. It can be methylated to creatine, which permits glycine and arginine to be used for creatine synthesis and promotes their applications in cell growth, division, signal transduction, etc. The other amino acids saved are used for muscle (LM, cardiac muscle) protein synthesis to increase the bound amino acid content.

The results of our study show that the amino acid profile in the heart and LM varied more than other visceral tissues, and this mainly stemmed from differences in tissue types. Skeletal muscle and cardiac muscle are striated muscles [47]. The kidney is the main excretory organ, and the liver is the largest gland and an important organ for material metabolism. The pancreas plays a key role in food digestion. The spleen contains a large amount of lymphoid tissue. In this experiment, the levels of bound amino acids in the liver, kidney, pancreas, and spleen were not affected by the concentration of GAA, coincident with our previous study [9]; that is, different concentrations of GAA had nothing significant on organ weight and protein content of finishing pigs. Tissue growth depends on protein synthesis and decomposition. GAA changed the physiological state of the body, but the balance of protein synthesis and decomposition was not disrupted; consequently, the weight of each organ did not change.

## 5. Conclusions

In this study, we suggested that GAA enhanced the plasma biochemical parameters, enhanced oxidative status, and ameliorated amino acid profiles of the heart and LM in finishing pigs. However, our current study only conveys viscera and LM, by which the impact of the gastrointestinal tract, brain, or different muscle types may need to be additionally investigated.

## Figures and Tables

**Table 1:** Effect of guanidine acetic acid (GAA) on carcass traits in finishing pigs ^1^.

Item	GAA, %				SEM	*p*-Value		
	0	0.05	0.10	0.15		ANOVA	Linear	Quadratic
Live weight, kg	129.17	130.67	129.5	130.33	1.70	0.919	0.898	1.000
Carcass weight, kg	92.58	93.64	94.03	95.43	1.55	0.674	0.671	0.334
Carcass yield, %	71.72	71.65	72.57	73.25	0.89	0.569	0.614	0.196
Back fat thickness, mm	35.39	33.33	36.00	32.86	1.61	0.487	0.517	0.750
Abdominal fat weight, kg	1.95	1.64	2.20	1.64	0.17	0.100	0.645	0.484
Loin muscle area, cm^2^	62.58	62.85	64.16	69.08	2.39	0.253	0.084	0.311

SEM = standard error of the mean. ^1^ Dietary treatments were: basal diet + 0, 0.05%, 0.10%, or 0.15% GAA, respectively. *n* = 6.

**Table 2:** Effect of guanidine acetic acid (GAA) on plasma biochemical parameters in finishing pigs ^1^.

Item	GAA, %				SEM	*p*-Value		
	0	0.05	0.10	0.15		ANOVA	Linear	Quadratic
Glucose, mmol/L	5.49 ^a^	4.77 ^ab^	4.47 ^b^	4.80 ^ab^	0.23	0.045	0.040	0.041
Total protein, g/L	72.72	79.26	70.39	77.14	2.66	0.185	0.751	0.973
Albumin, g/L	31.06 ^ab^	30.53 ^ab^	30.31 ^b^	34.01 ^a^	0.90	0.033	0.047	0.031
Creatinine, μmol/L	134.37	152.86	127.91	147.30	7.35	0.110	0.688	0.953
Uric acid, μmol/L	4.33	5.76	5.01	4.70	0.45	0.188	0.864	0.072
Urea, mmol/L	35.13	38.57	33.35	36.61	2.71	0.594	0.950	0.975
ALT, U/L	47.84	61.32	56.66	60.72	6.01	0.429	0.244	0.465
AST, U/L	24.22	27.60	26.27	27.65	2.70	0.805	0.483	0.726
Creatine kinase, U/L	975.00 ^b^	1170.50 ^ab^	1222.67 ^a^	1242.83 ^a^	52.97	0.008	0.002	0.116
GAA, mg/L	0.87 ^b^	1.17 ^ab^	1.33 ^a^	1.26 ^a^	0.08	0.006	0.003	0.045
Creatine, mg/L	20.16	20.70	22.45	22.50	0.69	0.060	0.011	0.731

SEM = standard error of the mean; ALT = alanine aminotransferase; AST = aspartate aminotransferase. ^1^ Dietary treatments were: basal diet + 0, 0.05%, 0.10%, or 0.15% GAA, respectively. *n* = 6. ^a,b^ Values within a row with different superscripts differ significantly at *p* < 0.05.

**Table 3:** Effect of guanidine acetic acid (GAA) on GAA and creatine contents in *longissimus thoracis* muscle (LM) and heart of finishing pigs (g/kg dry weight) ^1^.

Item	GAA, %				SEM	*p*-Value		
	0	0.05	0.10	0.15		ANOVA	Linear	Quadratic
GAA								
LM	49.47	46.57	49.20	50.35	2.44	0.779	0.666	0.462
heart	36.90	35.83	34.82	38.92	1.80	0.452	0.547	0.175
Creatine								
LM	14.89	14.93	15.48	15.33	0.16	0.067	0.014	0.562
heart	7.85	8.17	8.31	8.19	0.10	0.054	0.041	0.074

SEM = standard error of the mean. ^1^ Dietary treatments were: basal diet + 0, 0.05%, 0.10%, or 0.15% GAA, respectively. *n* = 6.

**Table 4:** Effect of guanidine acetic acid (GAA) on antioxidant capacity in finishing pigs ^1^.

Item	GAA, %				SEM	*p*-Value		
	0	0.05	0.10	0.15		ANOVA	Linear	Quadratic
Plasma								
MDA, nmol/L	1.59	1.24	1.09	0.98	0.18	0.152	0.030	0.545
Protein carbonyl, nmol/mL	1.03 ^a^	0.84 ^ab^	0.77 ^ab^	0.69 ^b^	0.06	0.010	0.001	0.395
SOD, U/mL	144.77 ^b^	212.47 ^a^	221.81 ^a^	232.60 ^a^	18.89	0.026	0.007	0.176
T-AOC, U/mL	5.68 ^b^	7.22 ^ab^	8.78 ^a^	8.01 ^ab^	0.60	0.011	0.005	0.069
GSH-Px, U/mL	744.75	693.43	824.37	782.4	77.44	0.720	0.519	0.956
Liver								
MDA, nmol/mgprot	0.58	0.57	0.45	0.44	0.09	0.592	0.215	0.981
Protein carbonyl, nmol/mgprot	1.18 ^a^	1.06 ^a^	1.02 ^ab^	0.83 ^b^	0.05	0.002	<0.001	0.531
SOD, U/mgprot	157.92 ^b^	180.82 ^ab^	199.23 ^ab^	219.48 ^a^	13.49	0.031	0.004	0.925
T-AOC, U/mgprot	0.66	0.68	0.77	0.62	0.11	0.804	0.965	0.437
GSH-Px, U/mgprot	49.45	60.45	50.53	54.62	3.93	0.310	0.779	0.443
Kidney								
MDA, nmol/mgprot	0.84	0.98	0.83	0.77	0.06	0.180	0.246	0.152
Protein carbonyl, nmol/mgprot	1.42 ^a^	1.34 ^ab^	1.23 ^bc^	1.19 ^c^	0.03	<0.001	<0.001	0.606
SOD, U/mgprot	74.38 ^b^	78.89 ^ab^	85.45 ^ab^	89.55 ^a^	3.04	0.011	0.001	0.948
T-AOC, U/mgprot	0.78	0.75	0.75	0.86	0.04	0.336	0.249	0.166
GSH-Px, U/mgprot	48.26	50.46	56.95	54.00	2.61	0.130	0.058	0.341
Pancreas								
MDA, nmol/mgprot	2.07	1.98	2.00	1.97	0.11	0.998	0.991	0.846
Protein carbonyl, nmol/mgprot	1.45 ^a^	1.43 ^ab^	1.25 ^ab^	1.23 ^b^	0.05	0.011	0.002	0.976
SOD, U/mgprot	47.4	47.21	48.78	49.12	2.00	0.875	0.464	0.896
T-AOC, U/mgprot	0.70	0.75	0.71	0.74	0.03	0.691	0.583	0.733
GSH-Px, U/mgprot	27.54	27.75	27.33	28.46	0.69	0.694	0.465	0.519
Spleen								
MDA, nmol/mgprot	0.93	0.83	0.83	0.80	0.03	0.087	0.025	0.326
Protein carbonyl, nmol/mgprot	1.44 ^a^	1.06 ^b^	0.90 ^b^	0.85 ^b^	0.06	<0.001	<0.001	0.015
SOD, U/mgprot	87.86 ^b^	96.89 ^a^	100.40 ^a^	99.35 ^a^	1.57	<0.001	<0.001	0.006
T-AOC, U/mgprot	0.82 ^b^	0.91 ^ab^	1.06 ^a^	1.06 ^a^	0.05	0.008	0.001	0.345
GSH-Px, U/mgprot	39.84 ^b^	44.86 ^a^	42.89 ^ab^	47.66 ^a^	1.17	0.002	0.001	0.923
Heart								
MDA, nmol/mgprot	0.31	0.30	0.30	0.27	0.01	0.230	0.068	0.532
Protein carbonyl, nmol/mgprot	1.79 ^a^	1.71 ^a^	1.29 ^b^	1.21 ^b^	0.07	<0.001	<0.001	0.945
SOD, U/mgprot	117.55 ^b^	127.59 ^ab^	129.63 ^a^	133.87 ^a^	2.71	0.003	<0.001	0.304
T-AOC, U/mgprot	0.44	0.48	0.50	0.52	0.02	0.119	0.020	0.613
GSH-Px, U/mgprot	5.51 ^b^	6.06 ^ab^	6.29 ^a^	6.34 ^a^	0.15	0.006	0.001	0.136
LM								
MDA, nmol/mgprot	0.12	0.05	0.07	0.06	0.02	0.096	0.077	0.176
Protein carbonyl, nmol/mgprot	2.00 ^a^	1.68 ^ab^	1.46 ^b^	1.13 ^c^	0.08	<0.001	<0.001	0.924
SOD, U/mgprot	85.15 ^b^	127.44 ^a^	142.57 ^a^	158.55 ^a^	8.65	<0.001	<0.001	0.177
T-AOC, U/mgprot	0.30	0.34	0.51	0.53	0.07	0.060	0.011	0.877
GSH-Px, U/mgprot	4.38 ^c^	4.74 ^bc^	5.13 ^b^	5.84 ^a^	0.11	<0.001	<0.001	0.135

SEM = standard error of the mean; MDA = malondialdehyde; SOD = superoxide dismutase; T-AOC = total antioxidant capacity; GSH-Px = glutathione peroxidase; LM = *longissimus thoracis* muscle. ^1^ Dietary treatments were: basal diet + 0, 0.05%, 0.10%, or 0.15% GAA, respectively. *n* = 6. ^a,b,c^ Values within a row with different superscripts differ significantly at *p* < 0.05.

**Table 5:** Effect of guanidine acetic acid (GAA) on the amino acid profile of heart in finishing pigs (% dry weight) ^1^.

Item	GAA, %				SEM	*p*-Value		
	0	0.05	0.10	0.15		ANOVA	Linear	Quadratic
Threonine	3.27	3.20	3.33	3.54	0.08	0.140	0.057	0.174
Valine	3.33	3.24	3.27	3.58	0.08	0.083	0.076	0.051
Methionine	2.00	1.78	1.80	2.02	0.07	0.092	0.782	0.016
Isoleucine	3.69	3.47	3.46	3.92	0.11	0.077	0.235	0.022
Leucine	6.73	6.47	6.60	7.22	0.16	0.063	0.072	0.033
Phenylalanine	3.10	3.03	3.12	3.32	0.07	0.108	0.058	0.100
Lysine	6.33	6.11	6.36	6.78	0.14	0.092	0.059	0.077
Histidine	2.02	1.98	2.03	2.17	0.05	0.130	0.062	0.127
Arginine	4.96	4.80	5.05	5.32	0.11	0.108	0.053	0.132
Proline	2.99	3.01	3.17	3.27	0.08	0.156	0.036	0.595
Serine	2.74	2.70	2.85	2.99	0.09	0.242	0.079	0.377
Glutamate	11.11	10.83	11.24	11.9	0.25	0.159	0.075	0.154
Glycine	3.80	3.87	4.12	4.16	0.13	0.277	0.073	0.970
Alanine	4.57	4.49	4.67	4.94	0.11	0.105	0.039	0.168
cystine	0.99	1.01	0.95	1.06	0.05	0.464	0.479	0.444
Aspartic acid	6.73	6.58	6.83	7.25	0.14	0.092	0.039	0.125
Tyrosine	2.72	2.55	2.63	2.84	0.06	0.098	0.198	0.032
EAA	35.44	34.06	35.00	37.86	0.81	0.075	0.071	0.044
NEAA	35.65	35.03	36.45	38.39	0.82	0.134	0.049	0.199
TAA	71.09	69.09	71.46	76.25	1.57	0.100	0.055	0.092

SEM = standard error of the mean; EAA = essential amino acids; NEAA = non-essential amino acids; TAA = total amino acids. ^1^ Dietary treatments were: basal diet + 0, 0.05%, 0.10%, or 0.15% GAA, respectively. *n* = 6.

**Table 6:** Effect of guanidine acetic acid (GAA) on the amino acid profile of *longissimus thoracis* muscle in finishing pigs ^1^.

Item	GAA, %				SEM	*p*-Value		
	0	0.05	0.10	0.15		ANOVA	Linear	Quadratic
Amino acid, % dry weight								
Threonine	4.26	4.19	4.46	4.34	0.08	0.147	0.791	0.048
Valine	4.36	4.41	4.60	4.44	0.10	0.439	0.512	0.226
Methionine	2.38 ^b^	2.52 ^ab^	2.63 ^a^	2.60 ^ab^	0.06	0.041	0.117	0.014
Isoleucine	4.45	4.68	4.90	4.75	0.02	0.516	0.657	0.022
Leucine	7.53	7.50	7.92	7.69	0.11	0.319	0.851	0.057
Phenylalanine	4.46	4.48	4.69	4.57	0.10	0.348	0.664	0.118
Lysine	8.56	8.47	8.97	8.73	0.18	0.277	0.977	0.088
Histidine	4.52	4.49	4.73	4.59	0.12	0.562	0.920	0.240
Arginine	5.82	5.80	6.14	5.96	0.13	0.242	0.831	0.076
Proline	3.08	3.08	3.37	3.12	0.06	0.189	0.662	0.067
Serine	3.45	3.31	3.57	3.48	0.07	0.143	0.341	0.068
Glutamate	13.81	13.75	14.49	14.04	0.31	0.346	0.853	0.133
Glycine	3.96	3.97	4.28	4.00	0.08	0.080	0.483	0.083
Alanine	5.48	5.48	5.92	5.60	0.11	0.169	0.703	0.073
cystine	1.70	1.67	1.66	1.62	0.09	0.936	0.928	0.093
Aspartic acid	8.87	8.93	9.42	9.06	0.19	0.242	0.916	0.057
Tyrosine	3.14	3.13	3.29	3.23	0.06	0.291	0.759	0.093
EAA	46.53	46.54	49.05	47.66	1.00	0.228	0.856	0.099
NEAA	43.55	43.25	45.81	44.15	0.88	0.257	0.803	0.094
TAA	90.08	89.79	94.85	91.80	1.87	0.257	0.249	0.482
Other, mg/g dry weight								
Carnosine	19.48 ^b^	20.63 ^b^	26.41 ^a^	24.34 ^a^	0.93	0.001	<0.001	0.164
Anserine	0.57 ^b^	0.70 ^ab^	0.80 ^a^	0.83 ^a^	0.06	0.029	0.004	0.447

SEM = standard error of the mean; EAA = essential amino acids; NEAA = non-essential amino acids; TAA = total amino acids. ^1^ Dietary treatments were: basal diet + 0, 0.05%, 0.10%, or 0.15% GAA, respectively. *n* = 6. ^a,b^ Values within a row with different superscripts differ significantly at *p* < 0.05.

## Data Availability

The data presented in this study are available in the article and supplementary material.

## Supplementary Materials

The following supporting information can be downloaded at: https://www.mdpi.com/article/10.3390/ani13101626/s1, Table S1: Effects of guanidinoacetic acid (GAA) on chemical compositions of longissimus thoracis muscle in finishing pigs; Table S2: Effect of guanidine acetic acid (GAA) on amino acid profiles of the liver in finishing pigs (% dry weight); Table S3: Effect of guanidine acetic acid (GAA) on amino acid profiles of the pancreas in finishing pigs (% dry weight); Table S4: Effect of guanidine acetic acid (GAA) on amino acid profile of spleen in finishing pigs (% dry weight); Table S5: Effect of guanidine acetic acid (GAA) on amino acid profile of kidney in finishing pigs (% dry weight).
