# Supplementary material for: Guanidine Acetic Acid Alters Tissue Bound Amino Acid Profiles and Oxidative Status in Finishing Pigs
Source: Animals (Basel). 2023 May 12;13(10):1626. doi: 10.3390/ani13101626 (PMC10215443; doi:10.3390/ani13101626)
Supplement: Supplementary file 1 [file animals-13-01626-s001.zip › animals-2249139-supplementary.pdf]

Table S1 Efects of guanidinoacetic acid (GAA) on chemical compositions of *longissimus thoracis* muscle in fnishing pigs.[1]

Item	GAA, %				SEM	*P*-value		
	0	0.05	0.10	0.15		ANOVA	Linear	Quadratic
Moisture, %	75.66	75.71	75.56	75.65	0.27	0.985	0.933	0.950
Crude protein, %	20.91	20.77	20.91	20.82	0.28	0.981	0.750	0.930
Ash, %	1.74	1.78	1.62	1.74	0.12	0.815	0.772	0.757
Lipid, %	2.03	1.85	1.71	1.60	0.21	0.560	0.254	0.886

[1] Results are presented as the mean and SEM (n= 6).

Table S2 Effect of guanidine acetic acid (GAA) on amino acid profiles of liver in finishing pigs (% dry weight). [1]

Item	GAA, %				SEM	*P*-value		
	0	0.05	0.10	0.15		ANOVA	Linear	Quadratic
Threonine	2.99	2.91	2.92	2.99	0.09	0.896	0.495	0.993
Valine	3.42	3.41	3.36	3.47	0.13	0.940	0.779	0.995
Methionine	1.55	1.49	1.47	1.55	0.09	0.890	0.522	0.879
Isoleucine	3.10	3.07	2.95	3.14	0.12	0.732	0.610	0.758
Leucine	6.38	6.24	6.23	6.34	0.25	0.967	0.651	0.927
Phenylalanine	3.32	3.26	3.26	3.31	0.11	0.974	0.672	0.966
Lysine	5.38	5.27	5.24	5.36	0.19	0.942	0.590	0.894
Histidine	2.05	1.97	2.02	2.03	0.10	0.956	0.594	0.897
Arginine	4.32	4.14	4.16	4.46	0.13	0.330	0.182	0.093
Proline	3.12	3.04	3.04	3.38	0.13	0.299	0.363	0.362
Serine	2.90	2.85	2.91	2.92	0.10	0.956	0.731	0.666
Glutamate	8.29	8.26	8.16	8.49	0.24	0.812	0.701	0.852
Glycine	4.03	4.10	4.09	4.65	0.23	0.368	0.788	0.282
Alanine	4.10	4.24	4.20	4.28	0.14	0.845	0.607	0.663
cystine	0.92	0.93	0.92	0.94	0.04	0.988	0.980	0.926
Aspartic acid	6.22	6.25	6.18	6.28	0.20	0.986	0.984	0.984
Tyrosine	2.50	2.30	2.35	2.46	0.11	0.534	0.157	0.951
EAA	32.50	31.75	31.59	32.63	1.13	0.887	0.524	0.988
NEAA	32.06	31.95	31.83	33.38	1.05	0.708	0.693	0.579
TAA	64.57	63.70	63.42	66.01	2.02	0.815	0.583	0.783

EAA= essential amino acids; NEAA = non-essential amino acids; TAA = total amino acids.

[1] Results are presented as the mean and SEM (n= 6).

Table S3 Effect of guanidine acetic acid (GAA) on amino acid profiles of pancreas in finishing pigs (% dry weight). [1]

Item	GAA, %				SEM	*P*-value		
	0	0.05	0.10	0.15		ANOVA	Linear	Quadratic
Threonine	2.63	2.76	2.64	2.98	0.19	0.568	0.302	0.589
Valine	2.91	3.01	2.97	3.27	0.19	0.571	0.746	0.609
Methionine	0.95	1.15	1.12	1.25	0.10	0.262	0.078	0.725
Isoleucine	2.78	2.93	2.92	3.25	0.21	0.501	0.172	0.690
Leucine	4.58	4.76	4.68	5.17	0.29	0.533	0.221	0.608
Phenylalanine	2.57	2.62	2.56	2.84	0.15	0.573	0.308	0.478
Lysine	4.00	4.07	3.99	4.42	0.22	0.511	0.261	0.439
Histidine	1.41	1.46	1.42	1.58	0.09	0.564	0.267	0.588
Arginine	3.68	3.75	3.62	4.08	0.22	0.501	0.309	0.397
Proline	2.64	2.70	2.61	2.91	0.16	0.602	0.352	0.497
Serine	2.83	3.00	2.84	3.25	0.23	0.582	0.319	0.627
Glutamate	6.50	6.75	6.58	7.32	0.41	0.505	0.228	0.559
Glycine	3.60	3.69	3.60	4.02	0.23	0.557	0.287	0.497
Alanine	3.22	3.31	3.21	3.57	0.20	0.585	0.312	0.521
cystine	1.19	1.20	1.21	1.28	0.07	0.828	0.415	0.682
Aspartic acid	5.91	6.11	5.97	6.68	0.44	0.624	0.297	0.581
Tyrosine	2.31	2.52	2.40	2.73	0.20	0.518	0.229	0.785
EAA	25.51	26.52	25.91	28.84	1.64	0.519	0.228	0.576
NEAA	28.19	29.28	28.42	31.76	1.90	0.568	0.276	0.574
TAA	53.70	55.80	54.33	60.59	3.54	0.544	0.252	0.574

EAA= essential amino acids; NEAA = non-essential amino acids; TAA = total amino acids.

[1] Results are presented as the mean and SEM (n= 6).

Table S4 Effect of guanidine acetic acid (GAA) on amino acid profile of spleen in finishing pigs (% dry weight). [1]

Item	GAA, %				SEM	*P*-value		
	0	0.05	0.10	0.15		ANOVA	Linear	Quadratic
Threonine	3.20	3.20	3.22	3.23	0.05	0.961	0.629	0.878
Valine	3.46	3.56	3.49	3.46	0.08	0.769	0.691	0.424
Methionine	1.60	1.69	1.67	1.70	0.06	0.657	0.321	0.680
Isoleucine	2.95	3.07	3.06	3.19	0.08	0.292	0.085	0.939
Leucine	6.37	6.52	6.49	6.50	0.14	0.900	0.636	0.650
Phenylalanine	3.09	3.10	3.08	3.07	0.05	0.975	0.767	0.822
Lysine	5.83	5.82	5.90	5.91	0.11	0.905	0.518	0.927
Histidine	2.15	2.19	2.18	2.12	0.05	0.765	0.565	0.380
Arginine	5.02	5.05	5.07	5.03	0.07	0.986	0.946	0.728
Proline	3.45	3.51	3.41	3.38	0.07	0.515	0.270	0.503
Serine	3.24	3.22	3.26	3.19	0.06	0.915	0.753	0.748
Glutamate	9.70	9.71	9.72	9.99	0.14	0.589	0.273	0.462
Glycine	4.77	4.90	4.71	4.61	0.12	0.384	0.191	0.396
Alanine	4.67	4.75	4.63	4.64	0.08	0.655	0.504	0.664
cystine	0.99	1.03	0.99	1.02	0.03	0.702	0.849	0.972
Aspartic acid	6.71	6.75	6.76	6.71	0.11	0.984	1.000	0.704
Tyrosine	2.54	2.62	2.65	2.65	0.05	0.657	0.262	0.591
EAA	33.68	34.19	34.16	34.22	0.59	0.937	0.635	0.736
NEAA	36.07	36.48	36.13	36.19	0.52	0.958	0.943	0.787
TAA	69.75	70.67	70.28	70.41	1.07	0.962	0.827	0.754

EAA= essential amino acids; NEAA = non-essential amino acids; TAA = total amino acids.

[1] Results are presented as the mean and SEM (n= 6).

Table S5 Effect of guanidine acetic acid (GAA) on amino acid profile of kidney in finishing pigs (% dry weight). [1]

Item	GAA, %				SEM	*P*-value		
	0	0.05	0.10	0.15		ANOVA	Linear	Quadratic
Threonine	2.96	2.97	3.05	3.13	0.06	0.296	0.069	0.660
Valine	3.18	3.19	3.23	3.34	0.08	0.482	0.167	0.494
Methionine	1.53	1.64	1.64	1.63	0.05	0.313	0.193	0.201
Isoleucine	3.16	3.17	3.26	3.37	0.09	0.345	0.090	0.594
Leucine	5.97	5.90	6.05	6.25	0.15	0.397	0.148	0.387
Phenylalanine	3.01	3.01	3.06	3.16	0.07	0.451	0.155	0.464
Lysine	4.85	4.86	4.95	5.09	0.11	0.460	0.135	0.616
Histidine	1.84	1.79	1.84	1.89	0.05	0.485	0.299	0.284
Arginine	4.61	4.81	4.76	4.85	0.10	0.439	0.184	0.629
Proline	3.35	3.62	3.63	3.51	0.12	0.487	0.484	0.176
Serine	2.95	3.00	3.07	3.10	0.06	0.367	0.086	0.882
Glutamate	8.51	8.80	8.87	9.06	0.20	0.329	0.079	0.811
Glycine	4.68	4.83	4.93	5.37	0.18	0.126	0.077	0.917
Alanine	4.10	4.31	4.19	4.29	0.09	0.376	0.320	0.563
cystine	1.06	1.06	1.08	1.08	0.02	0.692	0.293	0.901
Aspartic acid	6.04	6.08	6.15	6.34	0.14	0.463	0.142	0.581
Tyrosine	2.56	2.59	2.65	2.67	0.06	0.619	0.200	0.908
EAA	31.10	31.33	31.83	32.70	0.73	0.463	0.131	0.673
NEAA	33.24	34.82	34.47	34.98	0.67	0.350	0.163	0.466
TAA	64.34	66.15	66.30	67.68	1.38	0.462	0.134	0.889

EAA= essential amino acids; NEAA = non-essential amino acids; TAA = total amino acids.

[1] Results are presented as the mean and SEM (n= 6).
